# The Recognition of Solid Object Shape: The Importance of Inhomogeneity

**DOI:** 10.1177/2041669519870553

**Published:** 2019-08-13

**Authors:** J. Farley Norman, Sydney P. Wheeler, Lauren E. Pedersen, Lindsey M. Shain, Jonathan D. Kinnard, Joel Lenoir

**Affiliations:** Department of Psychological Sciences, Ogden College of Science and Engineering, Western Kentucky University, Bowling Green, KY, USA; Carol Martin Gatton Academy of Mathematics and Science, Bowling Green, KY, USA; Department of Psychological Sciences, Ogden College of Science and Engineering, Western Kentucky University, Bowling Green, KY, USA; College of Medicine, University of Kentucky, Lexington, KY, USA; Carol Martin Gatton Academy of Mathematics and Science, Bowling Green, KY, USA; School of Engineering & Applied Sciences, Ogden College of Science and Engineering, Western Kentucky University, Bowling Green, KY, USA

**Keywords:** three-dimensional perception, shape, visuo-haptic interactions, haptics/touch

## Abstract

A single experiment evaluated the haptic-visual cross-modal matching of solid object shape. One set of randomly shaped artificial objects was used (sinusoidally modulated spheres, SMS) as well as two sets of naturally shaped objects (bell peppers, *Capsicum annuum* and sweet potatoes, *Ipomoea batatas*). A total of 66 adults participated in the study. The participants’ task was to haptically explore a single object on any particular trial and subsequently indicate which of 12 simultaneously visible objects possessed the same shape. The participants’ performance for the natural objects was 60.9 and 78.7 percent correct for the bell peppers and sweet potatoes, respectively. The analogous performance for the SMS objects, while better than chance, was far worse (18.6 percent correct). All of these types of stimulus objects possess a rich geometrical structure (e.g., they all possess multiple *elliptic*, *hyperbolic*, and *parabolic* surface regions). Nevertheless, these three types of stimulus objects are perceived differently: Individual members of sweet potatoes and bell peppers are largely identifiable to human participants, while the individual SMS objects are not. Analyses of differential geometry indicate that these natural objects (e.g., bell peppers and sweet potatoes) possess heterogeneous spatial configurations of distinctly curved surface regions, and this heterogeneity is lacking in SMS objects. The current results therefore suggest that increases in surface structure heterogeneity facilitate human object recognition.

Most contemporary researchers believe that our visual and haptic perceptions of solid object shape depend upon *representations* (for discussions of potential representations of object shape, see Marr, 1982; Norman & Todd, 1992; Todd & Reichel, 1989); indeed, this is a fundamental assumption of cognitive science (e.g., Pylyshyn, 1980). Such representations of shape encode some type of *feature*. The Oxford English Dictionary defines feature (“Feature”, 2019) as “the elements which constitute bodily form” or “a distinctive or characteristic part of a thing.” According to mathematicians and differential geometers (e.g., Hilbert & Cohn-Vossen, 1983; Koenderink, 1984a, 1984b, 1990; Koenderink & van Doorn, 1992; Van Effelterre, 1994), characteristic parts of solid object surfaces include *elliptic*, *hyperbolic*, and *parabolic* regions. Concave elliptic regions are shaped like the inside of a bowl (i.e., are dimples), while convex elliptic regions are shaped like the outside of a bowl (i.e., are bumps). Hyperbolic surface regions are shaped like saddles—convex in one direction but concave in a perpendicular direction. Parabolic surface regions (shaped like a cylinder) separate saddles and bumps or saddles and dimples. Consider Figure 1, which shows a naturally shaped object (a bell pepper, *Capsicum annuum*) and a manmade solid object (a sinsusoidally modulated sphere) created by an algorithm developed by Norman, Todd, and Phillips (1995) and Todd and Norman (1995). It is obvious from an inspection of Figure 1 that the mathematicians are correct—both natural and manmade objects (at least smoothly curved ones) can be effectively and completely encoded in terms of these three generic types of surface regions. One aspect, however, that is not typically discussed—one that could potentially be quite important—is the spatial distribution of relevant surface features themselves.

**Figure 1.**
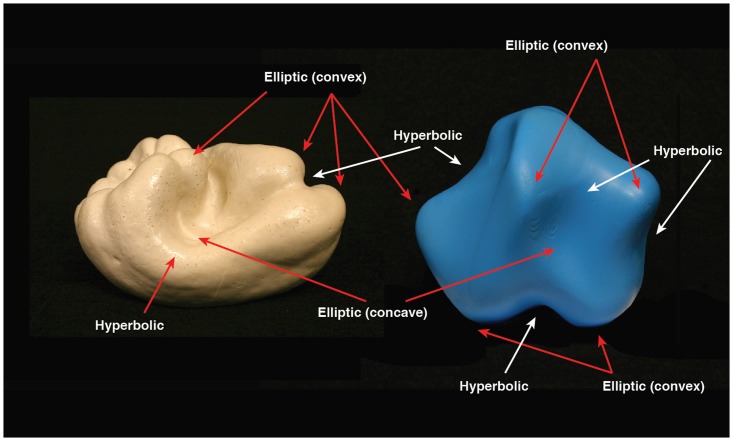
An illustration of the variously shaped surface regions (e.g., convex elliptic, concave elliptic, and hyperbolic) that exist on the surface of solid objects. Parabolic regions (locally shaped like a cylinder) separate elliptic and hyperbolic areas. The object portrayed on the left is a bell pepper (Bell Pepper 24), a naturally shaped object (*Capsicum annuum*), whereas the artificial object (a sinsuoidally modulated sphere, SMS 8) on the right was generated according to algorithms developed by Norman et al. (1995) and Todd and Norman (1995). Notice that the same distinctively curved surface regions (e.g., elliptic and hyperbolic) can effectively describe/represent the shapes of both natural and manmade object surfaces.

It is well known that visual and tactile inhomogeneities are salient (e.g., Koffka, 1935, pp. 110–127). Human sensory and perceptual systems do not respond well to homogeneous stimulation. Notice, for example, the high salience of a single blue dot against a red background (Wertheimer, 1923), the high salience of moving visual elements against a background of stationary elements (e.g., Metzger, 1936/2006, p. 58), or the easy tactile detection of a groove on an otherwise smooth surface. Might this general phenomenon (high perceptual sensitivity to inhomogeneities) extend to the perception and recognition of solid shape? The overall purpose of this study was to answer this question. To investigate this issue, we utilized a set of synthetic objects (sinusoidally modulated spheres, SMS) that were originally created by Norman et al. (1995) and Todd and Norman (1995). These objects are characterized by a high degree of homogeneity in the spatial distribution of their elliptic and hyperbolic surface regions (see Figure 2). In addition to the SMS objects, this study evaluated the recognizability of two types of natural objects (bell peppers and sweet potatoes), which are characterized by nonhomogeneous distributions of elliptic and hyperbolic surface regions (see Figures 3 and 4). All of these objects (SMS objects, bell peppers, and sweet potatoes) (a) have similar sizes and thus fit easily within the hands and (b) possess a rich collection of geometric surface structure (Koenderink, 1990; Koenderink & van Doorn, 1978). If the spatial distributions (homogeneous or nonhomogeneous) of surface features do not matter for human haptic and visual perception, then one would not necessarily expect significant differences in the ability to recognize SMS objects, bell peppers, and sweet potatoes. If spatial distributions of surface features are important for human perception, however, then one would perhaps expect a considerable superiority in object recognition for sets of natural objects whose members possess significant inhomogeneities in the distribution of important shape features (i.e., the elliptic and hyperbolic surface regions described by mathematicians and differential geometers).

**Figure 2.**
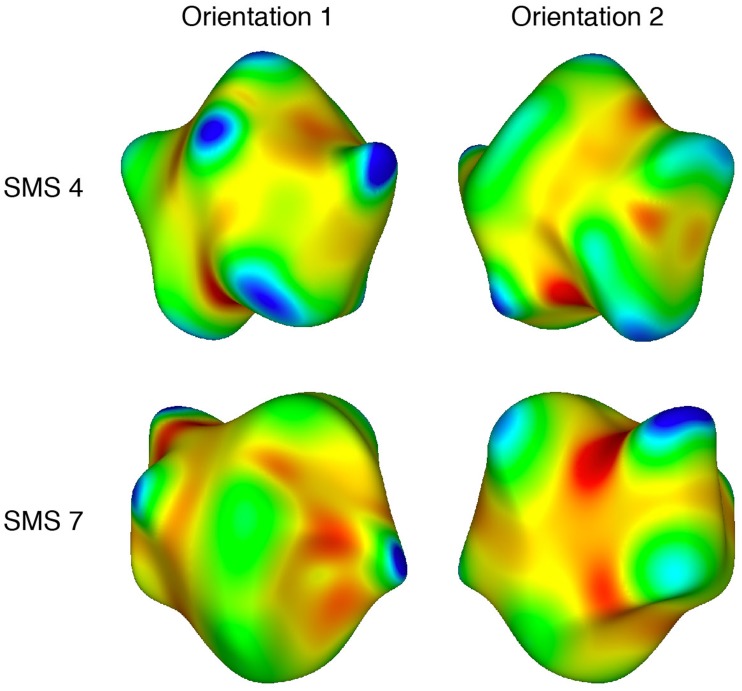
Plots of local shape (Gaussian curvature) for two of the sinusoidally modulated spheres (SMS objects) used as experimental stimuli; the two orientations shown are separated by a rotation of 180° around a Cartesian vertical (i.e., *y*) axis. Blue and green indicate surface areas that are locally *elliptic* (convex or concave), while red indicates surface areas that are *hyperbolic* (i.e., locally shaped like a saddle). Yellow indicates surface areas that are cylindrical (i.e., curved in one direction, but not in an orthogonal direction). This differential geometry was calculated using MeshLab (see Cignoni et al., 2008).

**Figure 3. fig3-2041669519870553:**
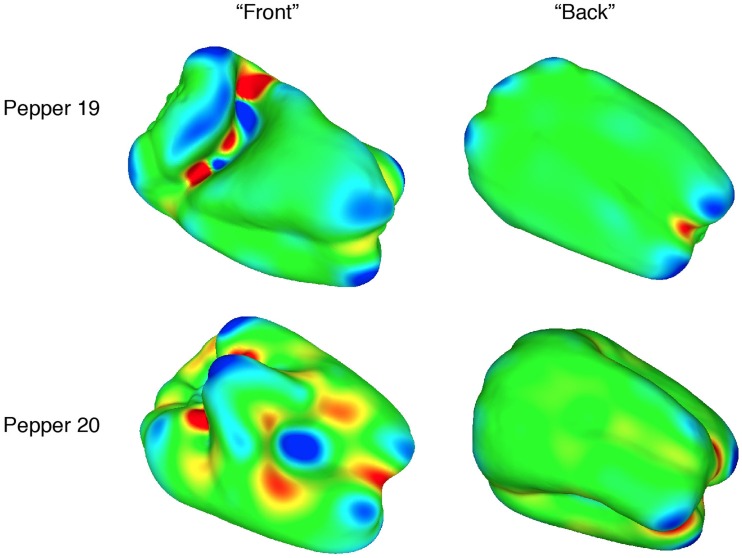
Plots of local shape (Gaussian curvature) for two of the bell peppers (*Capsicum annuum*) used as experimental stimuli. Blue and green indicate surface areas that are locally *elliptic* (convex or concave), while red indicates surface areas that are *hyperbolic* (i.e., locally shaped like a saddle). Yellow indicates surface areas that are cylindrical (i.e., curved in one direction, but not in an orthogonal direction). This differential geometry was calculated using MeshLab (see Cignoni et al., 2008).

**Figure 4.**
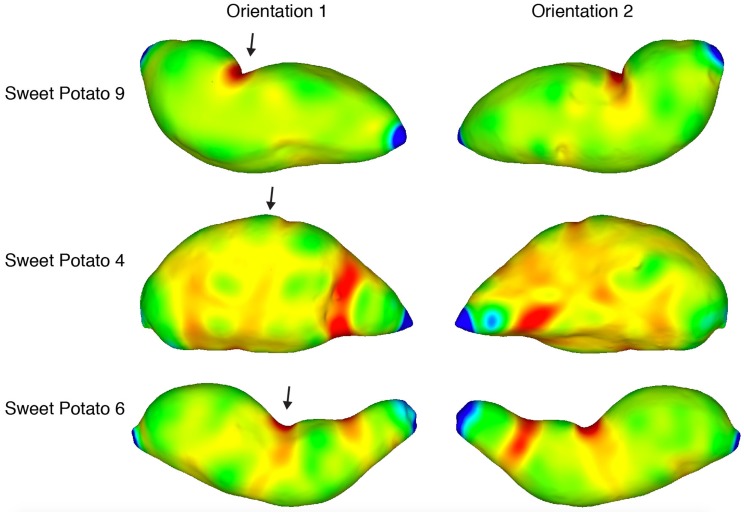
Plots of local shape (Gaussian curvature) for three of the sweet potatoes (*Ipomoea batatas*) used as experimental stimuli; the two orientations shown are separated by a rotation of 180° around a Cartesian vertical (i.e., *y*) axis. Blue and green indicate surface areas that are locally *elliptic* (convex or concave), while red indicates surface areas that are *hyperbolic* (i.e., locally shaped like a saddle). Yellow indicates surface areas that are cylindrical (i.e., curved in one direction, but not in an orthogonal direction). This differential geometry was calculated using MeshLab (see Cignoni et al., 2008). The arrows highlight the fact that two of these objects (Sweet Potatoes 6 and 9) have prominent saddle-shaped regions toward the middle of the object, while Sweet Potato 4 has elliptic (bump-like) regions in analogous areas.

## Method

### Experimental Stimuli and Apparatus

**Figure 5.**
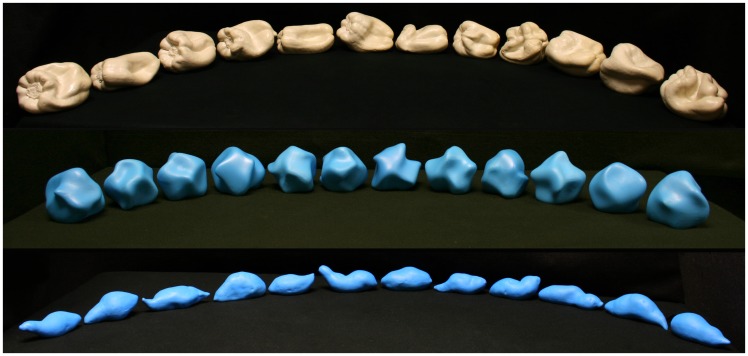
The three sets of experimental stimulus objects. The row of objects at top are naturally shaped bell peppers (*Capsicum annuum*), whereas the middle row of objects (sinusoidally modulated spheres, SMS) are manmade and were developed using an algorithm described by Norman et al., 2009). The bottom row shows the 12 sweet potato (*Ipomoea batatas*) replicas. The objects are arranged numerically from left to right (1–12 for SMS objects and sweet potatoes; 13–24 for bell peppers).

One group of stimulus objects consisted of 12 bell pepper (*Capsicum annuum*) replicas previously used in our research (Bell Peppers 13–24, see Norman et al., 2015, 2017). A second group of stimulus objects consisted of 12 SMS (SMS 1–12) that were randomly selected from a set of 1,000 objects used previously (Norman, Beers, Holmin, & Boswell, 2010; Norman, Swindle, Jennings, Mullins, & Beers, 2009). These SMS objects were then printed by authors J. F. N. and J. D. K. in PLA (polylactic acid) plastic using a Bits From Bytes three-dimensional (3D) Touch printer; the surfaces of the SMS objects used as visual stimuli were then smoothed using XTC-3D brush-on coating (Smooth-On, Inc.). The third group of stimuli used in this experiment were plastic (ABS, Acrylonitrile butadiene styrene) copies of 12 sweet potatoes (*Ipomoea batatas*) that were bought at grocery stores located in Bowling Green, KY, USA. Their shapes were scanned by the second author (S. P. W.) using a 3D laser scanner (NextEngine model 2020i Desktop 3D scanner). Following the scanning, the 12 sweet potato models were reduced in size by one third and were then printed by author J. L. using a Stratasys Dimension 3D printer. The surfaces of the sweet potatoes used as visual stimuli were then smoothed using XTC-3D brush-on coating (Smooth-On, Inc.). All three types of stimulus objects are shown in Figure 5. All objects possessed a similar size, at least when size is measured in terms of the maximum dimension/width (SMS objects: mean = 10.0 cm, *SD* = 0.33; Bell Peppers: mean = 13.1 cm, *SD* = 0.78; Sweet Potatoes: mean = 11.7 cm, *SD* = 1.21). In terms of volume, however, the sweet potatoes are smaller than the other types of objects, because their overall shape is cylindrical, instead of spherical (SMS objects: mean = 274.8 cm^3^, *SD* = 35.2; Bell Peppers: mean = 399.3 cm^3^, *SD* = 51.7; Sweet Potatoes: mean = 66.0 cm^3^, *SD* = 17.3). Notice that the SMS objects varied the least in terms of size (smallest *SD*) when size was measured in terms of maximum width, but it was the Sweet Potatoes whose size varied the least when it was measured in terms of volume. Two complete sets of each type of object (bell pepper, SMS, and sweet potato) were created to permit cross-modal (i.e., haptic-visual) matching. The order of stimulus presentations and collection of participant responses was performed by an Apple MacBook computer.

### Procedure

The procedure was similar to that developed by Gibson (1963) and used subsequently by Caviness (1964) and Norman, Norman, Clayton, Lianekhammy, and Zielke (2004; also see Norman, Phillips, et al., 2016). On any given trial, a participant would haptically explore a single randomly selected object (bell pepper, sweet potato, or SMS, depending upon participant) for 7 seconds using both hands (these haptically explored objects could not be seen, because of an occluding curtain). The participants’ task was to indicate which of 12 simultaneously visible objects (presented side by side on a tabletop, as shown in Figure 5) possessed the same (i.e., identical) shape. The participants could see the entire set of 12 visible objects while they were haptically exploring each stimulus object. Each participant made a total of 96 shape-matching judgments (8 trials for each of the 12 bell pepper, sweet potato, or SMS stimulus objects). The order of presentation of the stimulus objects was completely random.

### Participants

There were a total of 66 participants: 22 younger adults made shape-matching judgments for the bell peppers (mean age was 21.0 years, *SD* = 3.5; 14 were female), 22 younger adults made shape-matching judgments for the sweet potatoes (mean age was 20.2 years, *SD* = 2.4; 11 were female), and 22 younger adults made shape-matching judgments for the SMS objects (mean age was 22.4 years, *SD* = 3.2; 13 were female). All participants gave written consent prior to participation in the experiment. The experiment was approved by the Western Kentucky University Institutional Review Board. Our research was carried out in accordance with the Code of Ethics of the World Medical Association (Declaration of Helsinki). All participants were naïve regarding the purposes and motivations of the experiment. All participants had good visual acuity: The mean acuity measured at 1 m was −0.017 LogMAR (log minimum angle of resolution). Zero LogMAR indicates normal levels of visual acuity, while negative and positive values indicate better than normal acuity and worse than normal acuity, respectively.

## Results

**Figure 6.**
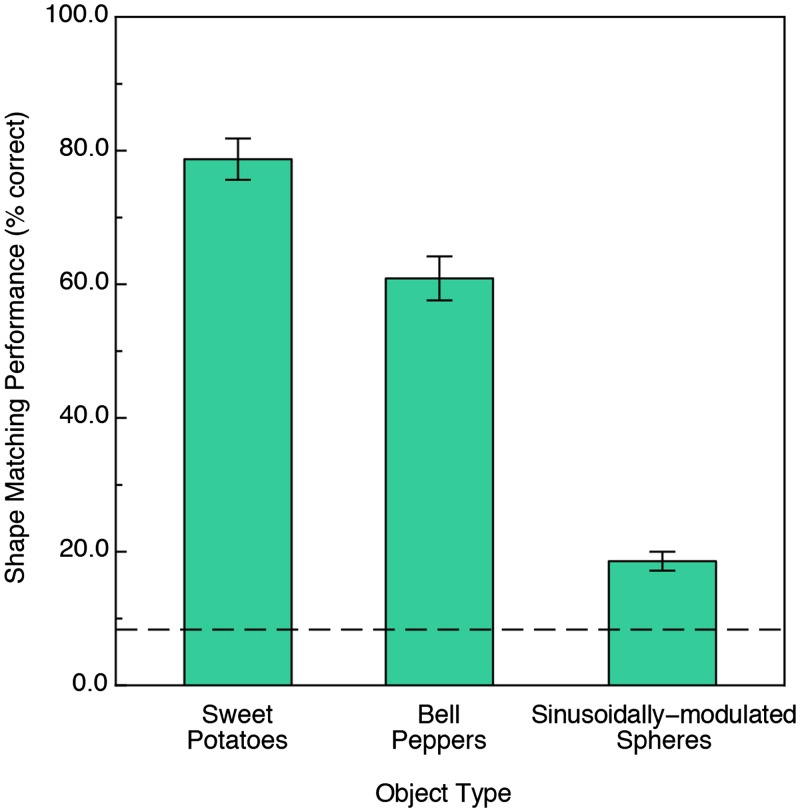
Experimental results. The participants’ cross-modal shape matching performance is plotted for all types of stimulus objects. The error bars indicate ± 1 *SE*. The dashed line indicates chance levels of performance.

The results are shown in Figure 6, which plots the participants’ shape-matching performance for all three types of stimulus object. It is easy to see that the performance obtained for the naturally shaped sweet potatoes and bell peppers was much higher (4.2 and 3.3 times higher, respectively) than that obtained for the SMS. A one-way between-subjects analysis of variance demonstrated that the effect of stimulus object type was significant, *F*(2, 63) = 128.6, *p* < .000001, ${\text{η}}_{\text{p}}^{\text{2}}$ = .80. Tukey HSD post hoc tests additionally showed that the performance obtained for each type of stimulus object was significantly different from all of the others (e.g., performance for the sweet potatoes was significantly higher than that obtained for the bell peppers, which was significantly higher than that obtained for the SMS objects). A one-sample *t* test demonstrated that while the overall performance obtained for the SMS objects was very low in absolute terms, it was significantly higher than chance, *t*(21) = 7.2, *p* < .000001.

Even though we expected that there might be significant differences in identification performance between the naturally shaped objects and the SMS objects (see introduction), we were nevertheless surprised at the magnitude of the difference (see Figure 6). To investigate the extremely poor performance for the SMS objects in more detail, we decided to ask several new participants (6) to perform the same matching task using unimodal vision or unimodal haptics. In all respects, the situation was analogous to that of the main experiment (which utilized haptic-visual matching). Three participants haptically explored a randomly selected SMS object on each trial for 7 seconds; they were then allowed to subsequently explore (also using haptics) the entire set of 12 SMS objects until they found one whose shape matched that of the original object. Another set of three participants performed the same task using only vision. On each trial, these participants viewed a randomly selected SMS object for 7 seconds that rotated continuously in depth about a Cartesian vertical axis. Afterwards, they looked at the entire set of 12 SMS objects until they found one that possessed the matching shape. These participants’ unimodal recognition ability was poor: The average performance was 25.0 percent correct (26.7 percent correct for unimodal vision and 23.3 percent correct for unimodal haptics). Although this unimodal performance for SMS objects was definitely poor, it was slightly higher than that obtained during cross-modal matching (see Figure 6). A one-sample *t* test, *t*(5) = 3.3, *p* = .022, two-tailed, revealed that this unimodal performance (25.0 percent correct) was significantly higher than that obtained during cross-modal matching (18.6 percent correct). Despite this numerical difference, it is clear that the SMS objects, while differing considerably in 3D shape from each other, are very difficult to recognize whether visually, haptically, or across modalities.

## Discussion

This study evaluated the ability of participants to perform cross-modal matching of solid object shape. A rich history of similar studies exists, beginning with those of Gibson (1963) and Caviness (1964). Similar investigations have continued forward until today (e.g., Abravanel, 1973; Davidson, Abbott, & Gershenfeld, 1974; Lacey & Campbell, 2006; Lacey, Peters, & Sathian, 2007; Lawson, 2009; Newell, Ernst, Tjan, & Bülthoff, 2001; Norman et al., 2017; Norman, Clayton, Norman, & Crabtree, 2008; Norman et al., 2004; Norman et al., 2016; Phillips, Egan, & Perry, 2009; Woods, O’Modhrain, & Newell, 2004). Our results extend this previous work and demonstrate that the type of object makes a tremendous difference in the ability of human participants to make cross-modal shape comparisons, even when all the objects in question have rich geometrical structure that is both visible and tangible.

**Figure 7. fig7-2041669519870553:**
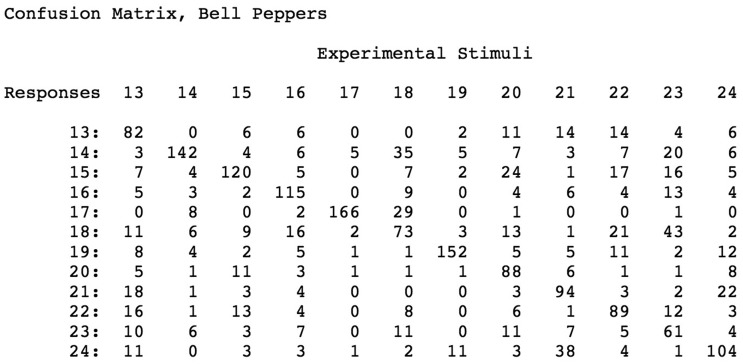
Confusion matrix obtained for bell pepper stimuli: response frequencies for each of the 12 stimulus objects. The frequencies of the correct responses are located along the diagonal.

**Figure 8. fig8-2041669519870553:**
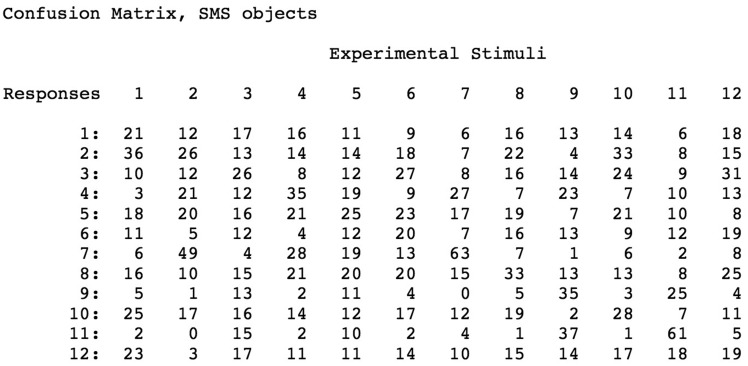
Confusion matrix obtained for SMS object stimuli: response frequencies for each of the 12 stimulus objects. The frequencies of the correct responses are located along the diagonal.

In this study, all of the objects’ geometrical structures (e.g., see Figures 2 [Fig fig3-2041669519870553]to 4) could be well described/represented using the distinctly curved surface regions (elliptic, hyperbolic, and parabolic) described by mathematicians. In Figures 2 [Fig fig3-2041669519870553]to 4, elliptic regions are colored green/blue, hyperbolic regions are colored red, and parabolic regions are indicated by yellow. Because our participants’ shape-matching performance (Figure 6) differed greatly depending upon whether the stimulus objects possessed homogeneous spatial distributions of elliptic and hyperbolic surface regions (SMS objects) or possessed inhomogeneities in the distribution of these regions (naturally shaped bell peppers and sweet potatoes), we propose the following. Any set of objects that have heterogeneous distributions of elliptic, hyperbolic, and parabolic regions will have members that are perceptually distinctive; in contrast, any set of objects that have homogeneous distributions of elliptic, hyperbolic, and parabolic regions will have members that are less perceptually distinctive (and are thus more confusable with each other). Consider Figure 3, which plots local surface shape for two of the bell peppers used in the experiment (the caps of both bell peppers, where they were originally attached to the plant, are located at the upper left). Notice that while both objects have plenty of rich geometrical structure, this structure is unevenly (heterogeneously) distributed. Both objects possess a *front* surface that has multiple elliptic and hyperbolic areas, but their back surfaces are essentially devoid of structure and are just *round* (all green). Notice furthermore that with regard to the front surface, all of *the action* (the variations in local surface shape) for Bell Pepper 19 occur toward the left side, whereas this is not true for Pepper 20. It is not surprising, therefore, that Bell Peppers 19 and 20 are perceptually distinguishable from each other (see the confusion matrix in Figure 7). Bell Pepper 19 is essentially round (blue/green) everywhere, except for one small crevice located on the front left (as depicted). In contrast, in the analogous location where Pepper 19 has an elliptical bump (lower right), Pepper 20 has an elliptic concavity or dimple (the large blue region surrounded by four hyperbolic/red regions). Now consider Figure 2, which similarly plots local surface shape for two of the SMS objects used in the experiment. Notice that the spatial arrangement of the elliptic and hyperbolic areas is quite different from that of the bell peppers: The variations in local surface shape occur everywhere and are homogeneously distributed across the object surfaces (note that there is no obvious *front* and *back* for SMS objects). Because the various elliptic and hyperbolic areas are homogeneously distributed across the SMS object surfaces, there is no asymmetry in arrangement (no inhomogeneity) to make one SMS object perceptually distinct from other SMS objects—according to this point of view, all SMS objects are highly similar and therefore SMS object pairs are perceptually more confusable than bell pepper object pairs (compare the confusion matrices shown in Figures 7 and 8).

**Figure 9.**
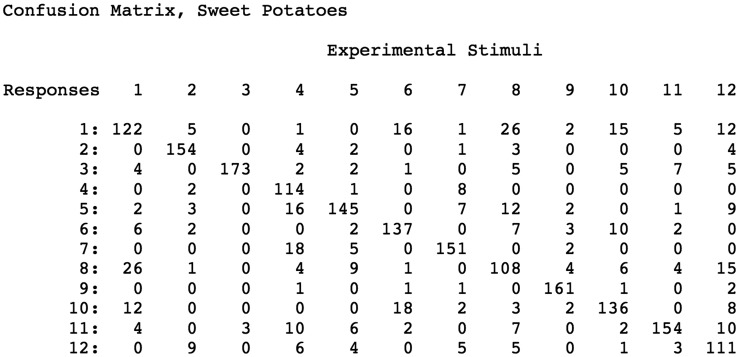
Confusion matrix obtained for Sweet Potato stimuli: response frequencies for each of the 12 stimulus objects. The frequencies of the correct responses are located along the diagonal.

Figure 4 plots local surface shape for the naturally shaped sweet potato (*Ipomoea batatas*) objects used as experimental stimuli. Although the overall shapes of the sweet potatoes are quite different from those of the bell peppers, there are some important similarities. Note, for example, that saddle-shaped regions (depicted in red) are highly localized on the sweet potato surfaces, and there are important differences between individual objects. Sweet Potatoes 6 and 9 have a prominent saddle (which is highly visible and tangible) toward the middle, while Sweet Potato 4 has a highly prominent convexity (convex elliptic) in the analogous position. This qualitative difference in overall shape (differential locations of saddles) makes Sweet Potatoes 4 and 6 perceptually distinct and highly discriminable (as well as Sweet Potatoes 4 and 9, see the confusion matrix presented in Figure 9). Notice that Sweet Potatoes 6 and 9 are also perceptually distinct (Figure 9), probably because of the differing number of prominent saddles: Sweet Potato 6 possesses two prominent saddles (which are both visible and tangible), while Sweet Potato 9 only has one.

**Figure 10.**
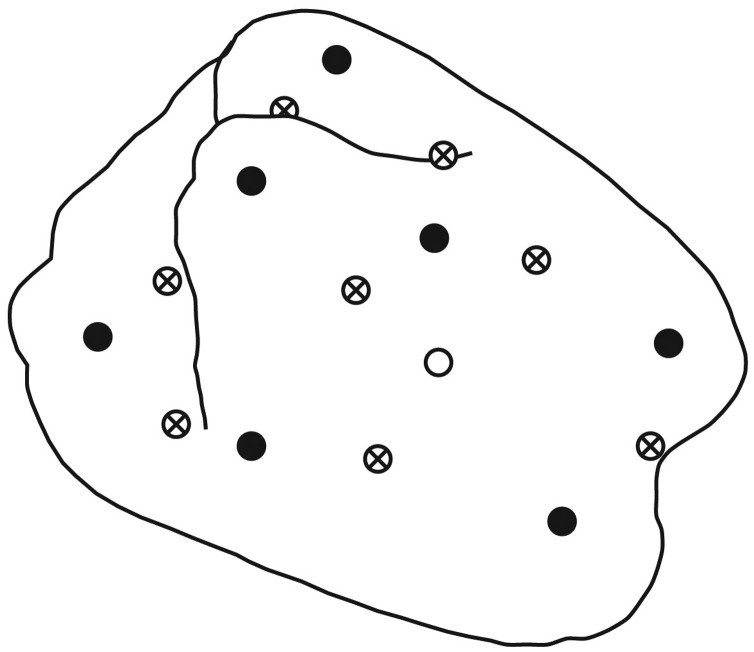
A plot of the occluding contour (Koenderink, 1984b) or occluding bound (Kennedy, 1974) of Bell Pepper 20, along with the relative locations of prominent hyperbolic surface regions (circles with crosses), concave elliptic regions (open circles), and convex elliptic regions (filled circles).

**Figure 11.**
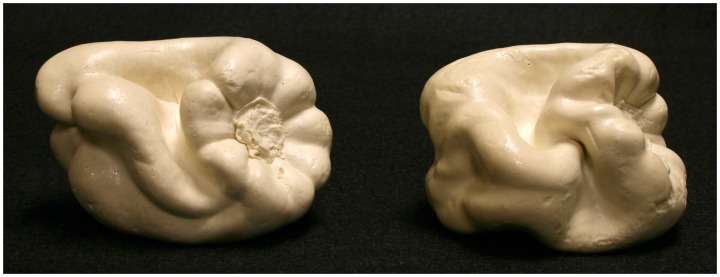
Photographs of Bell Peppers 21 (right) and 24 (left).

We believe that identifying an arbitrarily shaped solid object requires detecting a unique constellation of qualitative features as possibly stored in a representation such as that depicted in Figure 10. This figure plots Bell Pepper 20’s occluding contour (Koenderink, 1984b) or occluding bound (Kennedy, 1974) along with the spatial configuration (i.e., locations) of the nominal categories of local shape previously described (concave elliptic regions, open circles; convex elliptic regions, filled circles; hyperbolic or saddle regions, circles with crosses). It is important to note that it may not be necessary to explicitly represent parabolic (i.e., cylindrical) regions; parabolic regions are always located in between (i.e., they separate) elliptic and hyperbolic regions (e.g., see the yellow areas/contours depicted in Figure 3). We would expect that any two objects that have different spatial arrangements (or numbers) of saddles, concave elliptic regions, and convex elliptic regions would be discriminable to human participants (to some degree). We also predict that any two objects that have similar spatial arrangements of saddles, convexities, and concavities would be confusable, even if their local metric depths, surface slants, and curvature magnitudes are different. As examples, consider Bell Peppers 19 and 20 and 21 and 24. Bell Peppers 19 and 20 (Figures 3 and 7) are highly discriminable, because their spatial configurations of saddles, convexities, and concavities are quite different. In contrast, Bell Peppers 21 and 24 are confusable (see confusion matrix, Figure 7). Although their local metric quantities (surface depths, slants, and curvature magnitudes) obviously differ for corresponding locations (see Figure 11), they both possess a deep groove or trough toward the middle of the *front* surface. When participants haptically feel or see the trough of Bell Pepper 24, they often mistakenly identify that object as being Bell Pepper 21; likewise, when participants haptically feel or see the trough of Bell Pepper 21, they frequently misidentify that object as being Bell Pepper 24 (Figure 7). One advantage of a representation like that shown in Figure 10 is that it could be constructed for haptics as well as for vision, because these qualitative surface features (bumps, saddles, etc.) and their relative locations are tangible as well as visible. Therefore, this type of representation (see Koenderink & van Doorn, 1978; Lappin, Norman, & Phillips, 2011; Norman & Todd, 1992; Todd & Reichel, 1989) could subserve and permit successful cross-modal shape matching. At the moment, we (the authors) are unaware of any *specific* proposed encodings or representation for haptic solid shape, although abstract representations permitting haptic and cross-modal recognition do necessarily exist (e.g., see Easton, Greene, & Srinivas, 1997; Lacey, Pappas, Kreps, Lee, & Sathian, 2009; Lacey & Sathian, 2014; Lacey, Tal, Amedi, & Sathian, 2009). Research on vision has indicated that multiple representations of 3D shape simultaneously exist, and that different 3D shape primitives are represented with varying levels of precision (e.g., Norman & Todd, 1992; Norman & Todd, 1993; Norman & Todd, 1996; Norman & Todd, 1998; Norman, Todd, Norman, Clayton, & McBride, 2006; Todd & Reichel, 1989). Further research (e.g., Lacey, Pappas, et al., 2009) is needed to determine how many specific haptic and multimodal object representations exist and the exact nature of their contribution to the recognition of object shape.

## Conclusion

Given the current results (Figures 6 [Fig fig7-2041669519870553][Fig fig8-2041669519870553]to 9), we propose that the human visual and haptic systems possess sensitivity to variations in the spatial configuration (e.g., heterogeneity versus homogeneity) of the qualitatively distinct regions of local surface shape (*elliptic*, *hyperbolic*, and *parabolic*) described by Koenderink (1984a, 1984b, 1990) and Koenderink and van Doorn (1978).
